# Low NKp30, NKp46 and NKG2D expression and reduced cytotoxic activity on NK cells in cervical cancer and precursor lesions

**DOI:** 10.1186/1471-2407-9-186

**Published:** 2009-06-16

**Authors:** Trinidad Garcia-Iglesias, Alicia del Toro-Arreola, Benibelks Albarran-Somoza, Susana del Toro-Arreola, Pedro E Sanchez-Hernandez, Maria Guadalupe Ramirez-Dueñas, Luz Ma. Adriana Balderas-Peña, Alejandro Bravo-Cuellar, Pablo C Ortiz-Lazareno, Adrian Daneri-Navarro

**Affiliations:** 1 Departamento de Fisiología, Centro Universitario de Ciencias de la Salud, Universidad de Guadalajara, Sierra Mojada No 950, Colonia Independencia, Guadalajara, Jalisco, CP 44340, Mexico; 2 UMAE Hospital de Especialidades, Centro Medico Nacional de Occidente, Instituto Mexicano del Seguro Social, Mexico; 3 Centro de Investigacion Biomedica de Occidente, Instituto Mexicano del Seguro Social, Mexico

## Abstract

**Background:**

Persistent high risk HPV infection can lead to cervical cancer, the second most common malignant tumor in women worldwide. NK cells play a crucial role against tumors and virus-infected cells through a fine balance between activating and inhibitory receptors. Expression of triggering receptors NKp30, NKp44, NKp46 and NKG2D on NK cells correlates with cytolytic activity against tumor cells, but these receptors have not been studied in cervical cancer and precursor lesions. The aim of the present work was to study NKp30, NKp46, NKG2D, NKp80 and 2B4 expression in NK cells from patients with cervical cancer and precursor lesions, in the context of HPV infection.

**Methods:**

NKp30, NKp46, NKG2D, NKp80 and 2B4 expression was analyzed by flow cytometry on NK cells from 59 patients with cervical cancer and squamous intraepithelial lesions. NK cell cytotoxicity was evaluated in a 4 hour CFSE/7-AAD flow cytometry assay. HPV types were identified by PCR assays.

**Results:**

We report here for the first time that NK cell-activating receptors NKp30 and NKp46 are significantly down-regulated in cervical cancer and high grade squamous intraepithelial lesion (HGSIL) patients. NCRs down-regulation correlated with low cytolytic activity, HPV-16 infection and clinical stage. NKG2D was also down-regulated in cervical cancer patients.

**Conclusion:**

Our results suggest that NKp30, NKp46 and NKG2D down-regulation represent an evasion mechanism associated to low NK cell activity, HPV-16 infection and cervical cancer progression.

## Background

Cervical cancer is the second most common female malignant neoplasm worldwide. Human papillomavirus (HPV) is a necessary but not sufficient cause of cervical cancer. Co-factors that increase the risk for cervical cancer among HPV-DNA positive women, include oral contraceptives, smoking, high parity, previous sexually transmitted disease and immunodeficiency [1]. Cervical carcinogenesis implies HPV infection, viral persistence, progression and invasion [2]. Both innate and adaptive immune responses play a complex role against HPV infection. Spontaneous regression of high grade squamous intraepithelial lesions due to HPV-16 infection is associated with HPV-16 E7 peptide-specific CD4+ T-cell response and with lymphoproliferative responses to E2 plus IFN-gamma production [3-7]. However, innate immune response acts directly or indirectly against viral agents, through TLRs activation, dendritic cell presentation and NK cell function at cervical tissue level [8-11].

NK cells represent the first line of defense against viral pathogens, killing infected cells or via secretion of cytokines and chemokines [12]. There is also accumulating evidence for the crucial role of NK cells in tumor immunosurveillance [13]. NK cell activation and tumor lysis occur through a complex interaction between triggering receptors such as NKp30, NKp44, NKp46 and NKG2D with tumor cell ligands, in fine balance with inhibitory receptors and co-receptors [14]. Recently, it has been reported that activating NK cell receptor ligands MICA (NKG2D ligand) and CD155 (DNAM-1 ligand) are differentially expressed during the progression to cervical cancer [15]. However, the expression of NKp30, NKp46, NKG2D (triggering receptors) and co-receptors (NKp80 and 2B4) in NK cells from patients with cervical cancer and precursor lesions remains unknown. The aim of the present work was to study NKp30, NKp46, NKG2D, NKp80 and 2B4 expression in NK cells from patients with cervical cancer and precursor lesions, in the context of HPV infection.

## Methods

### Patients and samples

Blood samples were obtained from patients with invasive squamous cervical carcinoma (20), high grade squamous intraepithelial lesions (HGSIL) (20) and low grade squamous intraepithelial lesions (LGSIL) (19). Colposcopy and cytology results were confirmed by histopathology. Two different pathologists independently confirmed the diagnosis for all the specimens. Women without a history of abnormal Pap smears and negative for HPV were included as controls. Demographic, clinical characteristics and epidemiologic data were obtained. All women were attended at OPD Hospital Civil de Guadalajara, Mexico. The protocol was approved by Biomedicine Sciences and Ethic Committees (CSIM 200-22, 20000302032 and 2003, 259-0021), according to the last guidelines of the World Medical Association Declaration of Helsinki. Informed Consent was obtained from all participants enrolled in the study.

### Activating receptors (NKp30, NKp46 and NKG2D) and co-receptors (CD80 and 2B4) evaluation

Peripheral blood mononuclear cells (PBMC) were isolated by gradient Lymphoprep (Oslo Norway Nycomed™). NK cells were obtained by immunomagnetic negative selection (NK cell isolation kit, Miltenyi Biotec) in accordance with the manufacturer's instructions. After immunomagnetic depletion, NK cell CD56^+ ^were >95%, (confirmed by flow cytometry analysis). Cell concentration was adjusted to 1 × 10^5 ^and cells were incubated at 4°C for 30 min with proper dilution of anti-NKG2D (ECM217), anti-NKp30 (Z25); anti-NKp46 (BAB281), anti-NKp80 (MA252), or anti-2B4 (PP35) specific antibodies (kindly donated by Professor Alessandro Moretta, University of Genova, Italy). Cells were washed with a phosphate buffered saline (PBS) solution and incubated at 4°C for 30 min (in the dark) with FITC goat anti-mouse secondary Ab (IgG1). The cells were washed twice and incubated with 5 μL of PE-conjugated anti-CD56, PC5-conjugated anti-CD3 mAbs or with control isotype and fixed with 0.05% formaldehyde solution (all reagents from Beckman Coulter). The percentages and Median Fluorescence Intensity (MFI) were determined with a proper protocol and controls to compensate electronically for overlapping signals, using an EPICS XL-MCL flow cytometer (Beckman Coulter™).

### NK cell cytotoxicity assay

NK cell cytotoxicity against K562 cells was evaluated in a 4 hours CFSE/7-AAD flow cytometry assay [16]. NK cells were isolated from PBMC by cell sorting with anti-CD56 using FACSAria Cell Sorter (BD Bioscience). NK cell gate was set based on their forward/sideward light scatter and CD56/CD3 expression. The purity of the NK cells fraction was ≥ 98%. NK cells were labeled with 5–6-carboxyfluorescein diacetate succinimidyl ester (CSFE 200 nM) in PBS/1% BSA for 15 min at 37°C. CFSE-labeled NK cells were washed twice with PBS and seeded with a constant number of K562 cells (20,000) at different E:T ratios (1:1, 3:1, 10:1. 30:1). Target cells were incubated alone to measure basal cell death. Target cells and NK cells were incubated in complete medium for 4 hours in a 5% CO_2 _atmosphere at 37°C. Cells were washed twice in PBS-1% BSA containing 0.1% sodium azide (NaN_3_) and incubated in the same buffer plus 20 μL/mL to 7-amino actinomycin D (7-AAD, BD Biosciences) during 15 min at 4°C in darkness. Acquisition was performed with the FACS Diva Software (BD Bioscience). Cytotoxic activity was expressed as % Specific Lysis calculated by the following formula:

### DNA Extraction

DNA extraction was done in cervical cells obtained by cytobrush. Pellets containing cervical cells were obtained by centrifugation at 10 000 rpm for 3 min, 200 μg of Proteinase K was added for 48 hours at 37°C. Proteinase K was inactivated at 94°C for 10 minutes. Aqueous supernatant was transferred to another fresh microtube. DNA was precipitated by adding 100% ethanol and 20 mg/mL glycogen (Sigma) for 30 min at 22°C. The pellet was washed twice with 70% ethanol, dried, resuspended in 200 μL distilled water, and measured spectrophotometrically at 260/280 nm.

### PCR Assay

HPV typification was performed using specific primers (Table 1). All PCR reactions were performed in a total volume of 50 mL. PCR mixture contained 75 mM Tris-HCl pH 8.8, 20 mM (NH_4_)_2_SO_4_, 0.01% Tween 20, 2 mM MgCl2, 0.2 mM dNTPs, 0.6 mM of each primer, 1.25 U Taq DNA recombinant polymerase (Fermentas International Inc; Burlington, ON, Canada), and 100 ng DNA. Genomic DNA from SiHa (HPV16) and HeLa (HPV18) cells, tissue samples with known HPV infection for HPV6/11, HPV31, and 33, were used as positive controls. Genomic DNA from C33A cervical carcinoma cells was used as negative control. The cycling protocol for CpI/CPII was 94°C for 30 sec, 51°C for 30 sec, and 72°C for 60 sec for 40 cycles; HPV-16, 92°C for 120 sec, 48°C for 90 sec; HPV-6/11 and HPV-18, 92°C for 120 sec, 48°C for 90 sec, and 72°C for 60 sec for 38 cycles; and HPV-31 and HPV-33, 94°C for 60 sec, 45°C for 60 sec, and 72°C for 60 sec for 45 cycles. Amplification products were electrophoresed on a 1.8% agarose gel and visualized after ethidium bromide staining under UV light.

**Table 1:** Primer sequences for HPV typification

VIRUS	Primer Sequences (5'-3')	Amplimer length (base pair)
HVP (CpI/CpII)	(DNA) – CGTCCAAGAGGATACTGATC 1F (DNA) – GCACAGGGTCATAATAATGG 1R	188

HVP6/11	(DNA) – CTCTGCCGGTGGTCAGTGCAT 1F (DNA) – ATGCCTCCACGTCTGCAAC 1R	120

HVP16	(DNA) – CTGCACATGGGTGTGTGC 1F (DNA) – GCAGCTCTGTGCATAAC 1R	229

HVP18	(DNA) – GAATTCACTCTATGTGCAG 1F (DNA) – TAGTTGTTGCCTGTAGGTG 1R	221

HVP31	(DNA) – TTCAAAAATCCTGCAGAAAG 1F (DNA) – CTTTGACACGTTATACACCT 1R	320

HVP33	(DNA) – ACCTTTGCAACGATCTGAGG 1F (DNA) – GAACCGCAAACACAGTTTAC 1R	108

* w = a or t; s = c or g; y = c or t

### Statistical analysis

Statistical analysis was performed using the SPSS software package version 10.0 (SPSS, Inc Chicago, IL). Data were expressed as percentage, mean median fluorescence intensity (MFI) and analyzed by ANOVA. Significance was tested by non parametric test (Mann-Whitney U test). Differences were considered statistically significant when the *p *value was < 0.05. Spearman correlation was done to correlate activating receptors with HPV expression and Pearson correlation to cytotoxic activity.

## Results

The study enrolled 59 patients and 20 healthy women. Histological analysis confirmed that cervical cancer patients (20 cases) had squamous cell carcinomas (70% non-keratinizing large cell type and 30% keratinizing large cell type). HGSIL (20 cases) and LGSIL (19 cases) diagnoses were also confirmed by histopathology. There was no significant difference in mean age ± SD between cervical cancer patients (51.55 ± 14.55), HGSIL patients (41.29 ± 10.89), LGSIL patients (38.52 ± 9.72) and controls (44.10 ± 9.56).

Peripheral blood NK cell percentage was not significantly different between HGSIL, LGSIL and healthy women (mean ± SD = 18.31 ± 2.61, 17.84 ± 2.39 and 14.98 ± 3.41, respectively). However, patients with cervical cancer had significantly lower NK cell percentage (10.47 ± 3.58) in comparison with HGSIL patients (p < 0.05).

### Low NKp30, NKp46 and NKG2D expression on NK cells from patients with cervical cancer and HGSIL

**Figure 1:**
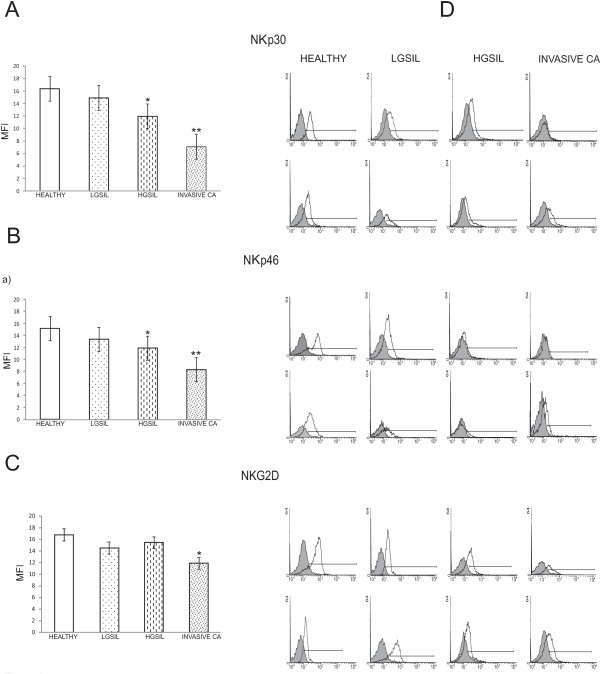
**NCRs and NKG2D expression are decreased in NK cells from cervical cancer patients**. NKp30, NKp46 and NKG2D expression was analyzed by flow cytometry on NK cells from patients with cervical cancer (20 cases), HGSIL (20 cases), LGSIL (19 cases) and 20 healthy women. MFI was expressed as mean ± SD. A) NKp30; B) NKp46: * HGSIL *versus *LGSIL and healthy women *p *< 0.02, ** Invasive Ca *versus *LGSIL and healthy women *p *< 0.0001; C) NKG2D: * Invasive Ca *versus *LGSIL and healthy women *p *< 0.02; D) Two representative histograms of each group are shown, filled curve: isotype control antibody.

We analyzed by flow cytometry the NCR (NKp30 and NKp46) phenotype (dull or bright), NKp30, NKp46 and NKG2D-expressing cells and MFI was also evaluated. Most healthy donors (90%) and patients with LGSIL (85%) had NK cells with NKp30^Bright ^and NKp46^bright ^phenotype. Patients with HGSIL had NK cells with NKp30^bright ^and NKp46^bright ^phenotype of 50%. In contrast, only 30% of patients with invasive cervical carcinoma showed NKp30^bright ^and NKp46^bright ^phenotype. We observed a significant decrease in NKp30 and NKp46 MFI on NK cells obtained from cervical cancer patients and HGSIL in comparison with healthy women and LGSIL patients (Figure 1). NKp30 and NKp46 expression (MFI) showed a significant negative correlation with HPV-16 infection (*r *= -0.508., *p *= 0.05). We did not find any significant correlation between NKp30 and NKp46 expression and other HPV types infection. Spearman's Correlation Coefficient between stage and NKp30 and NKp46 expression (MFI) showed a significant negative correlation (*r *= -0.774, *p *= 0.01).

We also found a significant lower expression of NKG2D on NK cells from cervical cancer patients in comparison with healthy women (p < 0.05). There was not a significant difference with HGSIL and LGSIL patients (Figure 1).

### Expression of co-receptors 2B4 and NKp80 in NK cells

**Figure 2:**
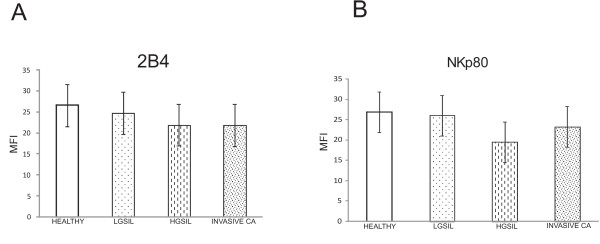
**2B4 and NKp80 co-receptor expression is not down-regulated on NK cells from cervical cancer patients**. 2B4 and NKp80 expression was analyzed by flow cytometry on NK cells from patients with cervical cancer (20 cases), HGSIL (20 cases), LGSIL (19 cases) and 20 healthy women. MFI was expressed as the mean ± SD. A) 2B4; B) NKp80; and C) Two representative histograms of each group are shown, filled curve: isotype control antibody.

Expression of co-receptors 2B4 and NKp80 (MFI) on NK cells was not significantly different between cervical cancer, HGSIL, LGSIL and healthy groups (Figure 2).

### Low cytotoxic activity in NK cells from cervical cancer and HGSIL patients

**Figure 3:**
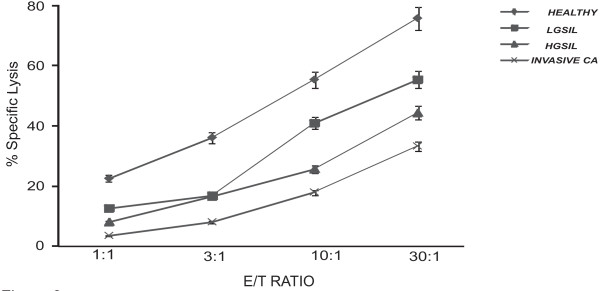
**NK cell specific lysis is decreased according to the natural history of cervical cancer: healthy women (6 controls) > LGSIL (6 cases) > HGSIL (5 cases) > cervical cancer (5 cases)**. NK cell cytotoxicity against K562 cells was evaluated in a 4 hour CFSE/7-AAD flow cytometry assay against K562 tumor line. Significant difference at 10:1 and 30:1 E:T ratios: Invasive Ca and HGSIL *versus *healthy women *p *< 0.0001 and *p *< 0.002, respectively.

NK cell-mediated specific cell lysis was decreased according to the natural history of cervical cancer: healthy women > LGSIL > HGSIL > cervical cancer (Figure 3). NK cell activity was significantly lower in patients with cervical carcinoma at all E/T ratios in comparison with LGSIL patients and healthy women (*p *< 0.0001). NK cell activity was also significantly decreased in HGSIL patients at 10:1 and 30:1 E/T ratios (*p *< 0.02) in comparison with LGSIL patients and healthy women (Figure 3). Percentage of specific lysis was correlated with NKp30 and NKp46 MFI. We found a significant correlation with NKp30 at 10:1 ratio (*r *= 0.686, *p *< 0.001) and at 30:1 ratio (r = 0.749, *p *< 0.001). We also found a significant correlation with NKp46 at 10:1 ratio (r = 0.468, *p *< 0.05) and at 30:1 ratio (r = 0.658, *p *< 0.01).

## Discussion

It has been proposed that immunosurveillance escape might be the seventh hallmark of cancer [17]. Intricate interactions between tumor and immune cells take place within the tumor microenvironment. These complex processes (often unknown) result in either tumor destruction (elimination phase), equilibrium phase or tumor growth (escape phase) by sculpting immunogenic cancer cells. Experimental and clinical evidence suggests that edition process dictates the immune response within tumor microenvironment. Cervical cancer represents an excellent opportunity to study the immune response and evasion mechanisms from precursor lesions to invasive carcinoma. NK cells play a crucial role in tumor immunosurveillance [13]. Arguments that may point to an important role of NK cells in the natural history of cervical cancer are: a) NK cells can recognize and either destroy virally infected cells or control the infection via cytokines (IFN-gamma and TNF-alpha), b) HPV-associated cervical tumors frequently exhibit a reduction in MHC class I expression as an immune evasion strategy, c) NK cell receptor ligands (MICA and CD155) are differentially expressed during progression to cervical cancer [15,18,19]. However, the biological significance of NK cells on HPV infection, viral persistence, SIL progression and invasion has not been addressed.

In this article we examined NKp30, NKp46, NKG2D, NKp80 and 2B4 expression in NK cells from patients with cervical cancer and precursor lesions, in the context of NK cell cytolytic activity and HPV infection. We report here for the first time that the NK cell-activating receptors NKp30 and NKp46 are significantly down-regulated in cervical cancer patients and HGSIL. Our results are consistent with previous reports in patients with myelocytic/monocytic acute myeloid leukemia. NK cells from such patients exhibit poor cytolytic functions due to deficient expression of NKp30, NKp44, and NKp46 receptors [20]. Myelodysplastic syndromes have reduced activating NK cell receptors (NKG2D and NKp30) in association with disease progression to myeloid leukemia [21]. It has been reported that NCR expression on NK cells correlates with the ability to lyse tumor cells [22]. NKp30 and NKp46 are the major NCRs against tumors. On the other hand, NK cell-mediated killing of HeLa cells was not significantly reduced with anti-NKp44 as seen with anti-NKp30 [23].

Our findings suggest a clinically relevant role of NCRs in cervical cancer progression, because low NKp30 and NKp46 expressions were correlated with diminished NK cell activity, HPV-16 infection and clinical stage. While the statistical correlation between diminished receptor expression and diminished specific lysis is strong, we cannot rule out the possibility that other mechanisms, such as lowered adhesin binding or decreased perforin B/granzyme activity might be operating in the reduction of specific lysis. To clarify this point, future experiments looking at changes of other molecules might be warranted. It is important to extend our findings using cervical cancer cell lines and fresh cervical cancer cells as targets. NCR ligands are still unknown, but by using soluble NCR fusion proteins the existence of NCR ligands on several tumor cells has been documented. Direct analysis of the expression of NKp30L and NKp40L on Hela cells (a cervical cancer cell line) revealed intracellular and extracellular expression of both ligands, wich was linked to NKp30-dependent lysis [23].

In this study we also found that the NK cell-activating receptor NKG2D is significantly down-regulated in cervical cancer patients. This result is consistent with a previous study reporting a significant decrease in the number of NKG2D-expressing NK and T cells in both cervical cancer and precursor lesion patients. However, they only found a significant correlation between high soluble MICA levels and low NKG2D in T cells [24].

NCRs and NKG2D may be down-regulated by different molecules and mechanisms such as TGF-beta, indoleamine 2,3-dioxygenase, prostaglandin E2, corticosteroids, 17 beta-estradiol and reactive oxygen species [25-29]. We do not know yet the precise mechanism that underlies NKp30, NKp46 and NKG2D down-regulation in NK cells from cervical cancer patients; however, it has been reported that TGF-beta1 mRNA overexpression is associated with progression from LGSIL to HGSIL [30,31]. TGF-beta is also upregulated at least two-fold in lymph node cervical cancer micrometastases [32]. Indoleamine 2,3-dioxygenase was also found at the invasion front of invasive cervical tumors and peritumoral stromal cells [33]. In the present work we found that NKp30 and NKp46 diminution was linked to HPV-16 infection. HPV-16 oncoproteins E6 and E7 down-modulate IFN-gamma and IL-18 response [34]. Other studies suggest that HPV infection affects dendritic cell migration and function [35,36]. We recently reported that CEACAM1, an adhesion molecule with NK cell inhibitory properties, is up-regulated in HGSIL linked to HPV-16 infection [37].

Taken together our results and previous reports suggest that NKp30, NKp46 and NKG2D down-regulation represent an evasion mechanism associated with low NK cell activity, HPV-16 infection and cervical cancer progression. Our findings may be important for development of new therapeutic strategies, for example the use of NCR-Ig fusion proteins with aim of inhibit tumor growth [38].

## Conclusion

Our results suggest that NKp30, NKp46 and NKG2D down-regulation represent an evasion mechanism associated with low NK cell activity, HPV-16 infection and cervical cancer progression.

## Competing interests

The authors declare that they have no competing interests.

## Authors' contributions

TGI: Performed the experimental work described in the study; searched and updated scientific literature and contributed scientific ideas and wrote draft manuscript. ATA: Was the core in the flow cytometry experiments and performed research; participated in the design of the study and reviewed the manuscript. BAS: Contributed to HPV detection and performed research. STA: Contributed in the design of the study; provided scientific ideas and assisted with the writing of the manuscript. PESH, MGRD and ABP contributed with scientific ideas and technical support. ABC and POL: Contributed with scientific ideas and NK cell cytotoxicity assay assistance. ADN: Conceived and designed the study; got the project Grant; coordinated the study; provided valuable scientific suggestions and reviewed, wrote and edited the manuscript (Corresponding Author). All authors read and approved the final manuscript.

## Pre-publication history

The pre-publication history for this paper can be accessed here:

http://www.biomedcentral.com/1471-2407/9/186/prepub

## References

[B1] Bosch FX de Sanjose S The epidemiology of human papillomavirus infection and cervical cancer Dis Markers 2007 23 4 213 227 17627057 10.1155/2007/914823 PMC3850867

[B2] Schiffman M Castle PE Jeronimo J Rodriguez AC Wacholder S Human papillomavirus and cervical cancer Lancet 2007 370 9590 890 907 10.1016/S0140-6736(07)61416-0 17826171

[B3] Kobayashi A Greenblatt RM Anastos K Minkoff H Massad LS Young M Levine AM Darragh TM Weinberg V Smith-McCune KK Functional attributes of mucosal immunity in cervical intraepithelial neoplasia and effects of HIV infection Cancer Res 2004 64 18 6766 6774 10.1158/0008-5472.CAN-04-1091 15374995

[B4] Frazer I Correlating immunity with protection for HPV infection Int J Infect Dis 2007 11 Suppl 2 S10 16 10.1016/S1201-9712(07)60016-2 18162240

[B5] Stanley M Immunobiology of HPV and HPV vaccines Gynecol Oncol 2008 109 2 Suppl S15 21 10.1016/j.ygyno.2008.02.003 18474288

[B6] Peng S Trimble C Wu L Pardoll D Roden R Hung CF Wu TC HLA-DQB1*02-restricted HPV-16 E7 peptide-specific CD4+ T-cell immune responses correlate with regression of HPV-16-associated high-grade squamous intraepithelial lesions Clin Cancer Res 2007 13 8 2479 2487 10.1158/1078-0432.CCR-06-2916 17438108 PMC3181117

[B7] Dillon S Sasagawa T Crawford A Prestidge J Inder MK Jerram J Mercer AA Hibma M Resolution of cervical dysplasia is associated with T-cell proliferative responses to human papillomavirus type 16 E2 J Gen Virol 2007 88 Pt 3 803 813 10.1099/vir.0.82678-0 17325352

[B8] Wira CR Fahey JV Sentman CL Pioli PA Shen L Innate and adaptive immunity in female genital tract: cellular responses and interactions Immunol Rev 2005 206 306 335 10.1111/j.0105-2896.2005.00287.x 16048557

[B9] Manickam A Sivanandham M Tourkova IL Immunological role of dendritic cells in cervical cancer Adv Exp Med Biol 2007 601 155 162 17713002 10.1007/978-0-387-72005-0_16

[B10] Hasan UA Bates E Takeshita F Biliato A Accardi R Bouvard V Mansour M Vincent I Gissmann L Iftner T TLR9 expression and function is abolished by the cervical cancer-associated human papillomavirus type 16 J Immunol 2007 178 5 3186 3197 17312167 10.4049/jimmunol.178.5.3186

[B11] McKenzie J King A Hare J Fulford T Wilson B Stanley M Immunocytochemical characterization of large granular lymphocytes in normal cervix and HPV associated disease J Pathol 1991 165 1 75 80 10.1002/path.1711650112 1659628

[B12] Lanier LL Evolutionary struggles between NK cells and viruses Nat Rev Immunol 2008 8 4 259 268 18340344 10.1038/nri2276 PMC2584366

[B13] Waldhauer I Steinle A NK cells and cancer immunosurveillance Oncogene 2008 27 45 5932 5943 10.1038/onc.2008.267 18836474

[B14] Moretta L Bottino C Pende D Castriconi R Mingari MC Moretta A Surface NK receptors and their ligands on tumor cells Semin Immunol 2006 18 3 151 158 10.1016/j.smim.2006.03.002 16730454

[B15] Textor S Durst M Jansen L Accardi R Tommasino M Trunk MJ Porgador A Watzl C Gissmann L Cerwenka A Activating NK cell receptor ligands are differentially expressed during progression to cervical cancer Int J Cancer 2008 123 10 2343 2353 10.1002/ijc.23733 18712710

[B16] Lecoeur H Fevrier M Garcia S Riviere Y Gougeon ML A novel flow cytometric assay for quantitation and multiparametric characterization of cell-mediated cytotoxicity J Immunol Methods 2001 253 1–2 177 187 10.1016/S0022-1759(01)00359-3 11384679

[B17] Bui JD Schreiber RD Cancer immunosurveillance, immunoediting and inflammation: independent or interdependent processes? Curr Opin Immunol 2007 19 2 203 208 10.1016/j.coi.2007.02.001 17292599

[B18] Strowig T Brilot F Munz C Noncytotoxic functions of NK cells: direct pathogen restriction and assistance to adaptive immunity J Immunol 2008 180 12 7785 7791 18523242 10.4049/jimmunol.180.12.7785 PMC2575662

[B19] Bottley G Watherston OG Hiew YL Norrild B Cook GP Blair GE High-risk human papillomavirus E7 expression reduces cell-surface MHC class I molecules and increases susceptibility to natural killer cells Oncogene 2008 27 12 1794 1799 10.1038/sj.onc.1210798 17828295

[B20] Fauriat C Just-Landi S Mallet F Arnoulet C Sainty D Olive D Costello RT Deficient expression of NCR in NK cells from acute myeloid leukemia: Evolution during leukemia treatment and impact of leukemia cells in NCRdull phenotype induction Blood 2007 109 1 323 330 10.1182/blood-2005-08-027979 16940427

[B21] Epling-Burnette PK Bai F Painter JS Rollison DE Salih HR Krusch M Zou J Ku E Zhong B Boulware D Reduced natural killer (NK) function associated with high-risk myelodysplastic syndrome (MDS) and reduced expression of activating NK receptors Blood 2007 109 11 4816 4824 17341666 10.1182/blood-2006-07-035519 PMC1885518

[B22] Sivori S Pende D Bottino C Marcenaro E Pessino A Biassoni R Moretta L Moretta A NKp46 is the major triggering receptor involved in the natural cytotoxicity of fresh or cultured human NK cells. Correlation between surface density of NKp46 and natural cytotoxicity against autologous, allogeneic or xenogeneic target cells Eur J Immunol 1999 29 5 1656 1666 10.1002/(SICI)1521-4141(199905)29:05<1656::AID-IMMU1656>3.0.CO;2-1 10359120

[B23] Byrd A Hoffmann SC Jarahian M Momburg F Watzl C Expression analysis of the ligands for the Natural Killer cell receptors NKp30 and NKp44 PLoS ONE 2007 2 12 e1339 18092004 10.1371/journal.pone.0001339 PMC2129109

[B24] Arreygue-Garcia NA Daneri-Navarro A del Toro-Arreola A Cid-Arregui A Gonzalez-Ramella O Jave-Suarez LF Aguilar-Lemarroy A Troyo-Sanroman R Bravo-Cuellar A Delgado-Rizo V Augmented serum level of major histocompatibility complex class I-related chain A (MICA) protein and reduced NKG2D expression on NK and T cells in patients with cervical cancer and precursor lesions BMC Cancer 2008 8 16 18208618 10.1186/1471-2407-8-16 PMC2270854

[B25] Li H Han Y Guo Q Zhang M Cao X Cancer-expanded myeloid-derived suppressor cells induce anergy of NK cells through membrane-bound TGF-beta1 J Immunol 2009 182 1 240 249 19109155 10.4049/jimmunol.182.1.240

[B26] Spaggiari GM Capobianco A Abdelrazik H Becchetti F Mingari MC Moretta L Mesenchymal stem cells inhibit natural killer-cell proliferation, cytotoxicity, and cytokine production: role of indoleamine 2,3-dioxygenase and prostaglandin E2 Blood 2008 111 3 1327 1333 10.1182/blood-2007-02-074997 17951526

[B27] Vitale C Chiossone L Cantoni C Morreale G Cottalasso F Moretti S Pistorio A Haupt R Lanino E Dini G The corticosteroid-induced inhibitory effect on NK cell function reflects down-regulation and/or dysfunction of triggering receptors involved in natural cytotoxicity Eur J Immunol 2004 34 11 3028 3038 10.1002/eji.200425418 15368269

[B28] Hao S Zhao J Zhou J Zhao S Hu Y Hou Y Modulation of 17beta-estradiol on the number and cytotoxicity of NK cells in vivo related to MCM and activating receptors Int Immunopharmacol 2007 7 13 1765 1775 10.1016/j.intimp.2007.09.017 17996687

[B29] Romero AI Thoren FB Brune M Hellstrand K NKp46 and NKG2D receptor expression in NK cells with CD56dim and CD56bright phenotype: regulation by histamine and reactive oxygen species Br J Haematol 2006 132 1 91 98 10.1111/j.1365-2141.2005.05842.x 16371024

[B30] Lee JC Lee KM Kim DW Heo DS Elevated TGF-beta1 secretion and down-modulation of NKG2D underlies impaired NK cytotoxicity in cancer patients J Immunol 2004 172 12 7335 7340 15187109 10.4049/jimmunol.172.12.7335

[B31] Baritaki S Sifakis S Huerta-Yepez S Neonakis IK Soufla G Bonavida B Spandidos DA Overexpression of VEGF and TGF-beta1 mRNA in Pap smears correlates with progression of cervical intraepithelial neoplasia to cancer: implication of YY1 in cervical tumorigenesis and HPV infection Int J Oncol 2007 31 1 69 79 17549406

[B32] Hagemann T Bozanovic T Hooper S Ljubic A Slettenaar VI Wilson JL Singh N Gayther SA Shepherd JH Van Trappen PO Molecular profiling of cervical cancer progression Br J Cancer 2007 96 2 321 328 10.1038/sj.bjc.6603543 17242701 PMC2360010

[B33] Sedlmayr P Semlitsch M Gebru G Karpf E Reich O Tang T Wintersteiger R Takikawa O Dohr G Expression of indoleamine 2,3-dioxygenase in carcinoma of human endometrium and uterine cervix Adv Exp Med Biol 2003 527 91 95 15206720 10.1007/978-1-4615-0135-0_10

[B34] Lee SJ Cho YS Cho MC Shim JH Lee KA Ko KK Choe YK Park SN Hoshino T Kim S Both E6 and E7 oncoproteins of human papillomavirus 16 inhibit IL-18-induced IFN-gamma production in human peripheral blood mononuclear and NK cells J Immunol 2001 167 1 497 504 11418688 10.4049/jimmunol.167.1.497

[B35] Guess JC McCance DJ Decreased migration of Langerhans precursor-like cells in response to human keratinocytes expressing human papillomavirus type 16 E6/E7 is related to reduced macrophage inflammatory protein-3alpha production J Virol 2005 79 23 14852 14862 16282485 10.1128/JVI.79.23.14852-14862.2005 PMC1287574

[B36] Jimenez-Flores R Mendez-Cruz R Ojeda-Ortiz J Munoz-Molina R Balderas-Carrillo O de la Luz Diaz-Soberanes M Lebecque S Saeland S Daneri-Navarro A Garcia-Carranca A High-risk human papilloma virus infection decreases the frequency of dendritic Langerhans' cells in the human female genital tract Immunology 2006 117 2 220 228 16423058 10.1111/j.1365-2567.2005.02282.x PMC1782223

[B37] Albarran-Somoza B Franco-Topete R Delgado-Rizo V Cerda-Camacho F Acosta-Jimenez L Lopez-Botet M Daneri-Navarro A CEACAM1 in cervical cancer and precursor lesions: association with human papillomavirus infection J Histochem Cytochem 2006 54 12 1393 1399 10.1369/jhc.6A6921.2006 16924126 PMC3958116

[B38] Arnon TI Markel G Bar-Ilan A Hanna J Fima E Benchetrit F Galili R Cerwenka A Benharroch D Sion-Vardy N Harnessing soluble NK cell killer receptors for the generation of novel cancer immune therapy PLoS ONE 2008 3 5 e2150 18478075 10.1371/journal.pone.0002150 PMC2364651

